# USP7 regulates growth and maintains the stemness of p53-mutant colorectal cancer cells via stabilizing of mutant p53

**DOI:** 10.3389/fonc.2024.1427663

**Published:** 2024-09-12

**Authors:** Xue Li, Jie Pan, Pengcheng Zheng

**Affiliations:** ^1^ Department of Pharmacy, The First People’s Hospital of Yunnan Province, Kunming, Yunnan, China; ^2^ The Affiliated Hospital of Kunming University of Science and Technology, Kunming, Yunnan, China; ^3^ Department of Stomatology, The First People’s Hospital of Yunnan Province, Kunming, Yunnan, China

**Keywords:** USP7, stemness, mutant p53, P5091, colorectal cancer, antitumor

## Abstract

**Introduction:**

TP53 is one of the most frequently mutated genes among all cancers, and TP53 mutants occur more than 40% in colorectal cancers (CRCs). Accumulation of mutant p53 may augment colorectal cancer stem cells (CCSCs) phenotype and enhance colorectal tumorigenesis. Thus, reducing the level of mutant p53 protein is an attractive anticancer strategy.

**Methods:**

CSC-enriched cancer cells were obtained by tumor sphere formation assay. The effects of USP7 on the proliferation of cancer cells were determined by MTS and colony formation assays. Wound healing assay was used to test cell migratory abilities. qPCR and western blotting assays were performed to verify the mRNA and protein levels of CSC markers, USP7 and p53. Co-immunoprecipitation assay was used to test the interaction effects between USP7 and p53.

**Results:**

In this study, we found that USP7 and mutant p53 were dramatically elevated in CSC-enriched colorectal cancer cells and USP7 expression was positively associated with self-renewal and maintenance of CCSCs. USP7 regulated cell growth, stemness and migration of colorectal cancer cells. USP7 depletion significantly reduced proliferation of cancer cells and suppressed the self-renewal of CSC-enriched colorectal cancer cells. Further studies indicated that USP7 knockdown could significantly decrease mutant p53 protein levels both in CRCs and CSC-enriched colorectal cancer cells. Moreover, mutant p53 was stabilized by USP7 and they interacted with each other. Furthermore, USP7 inhibitor P5091 also diminished CCSCs self-renewal and reduced mutant p53 levels.

**Conclusion:**

Taken together, our findings demonstrated that USP7 involved in the modulation of CCSCs stemness, as well as a critical target for clinical treatment of cancers with different p53 mutations.

## 1 Introduction

Although colorectal cancer has been studied for years, it is still the third highest prevalence and the second most common cause of death among all cancer types. In 2023, colorectal cancer accounted for approximately 8% of all incident cases and 8-9% of total deaths worldwide (1). Despite many improvements in CRC treatment, tumor recurrence and metastases prompt us to explore novel therapeutic strategies which can improve the clinical outcomes.

It is now well accepted that cancer stem cells (CSCs), a small subgroup of tumor cells, represent an important target for anticancer therapeutics (2). CSCs possess many stem cell properties, including immortal and self-renewal, which drive tumor initiation, maintenance, metastasis, and recurrence (3). Colorectal cancer stem cells were discovered and proved to express high levels of CD133, CD166, CD44, OCT4 and NANOG (4). It was observed that CCSCs are resistant to conventional chemotherapies and radiotherapies (5, 6). Thus, cancer stem cell-based therapy has opened a new window to overcome the therapeutic refractoriness (3).

It has been reported that ubiquitin-specific peptidase 7 (USP7), one of the most widely studies deubiquitinases (DUBs), plays a critical role in stem cell maintenance and differentiation (7–9). USP7 was found to regulate many crucial cell processes, including cell growth, cell cycle, DNA repair and tumorigenesis (10). Previous studies demonstrated that USP7 protein level was elevated in CRC cells, particularly in CCSCs. USP7 knockdown reduced the sphere formation ability of HT29 and HCT116 cells (8). USP7 has many reported substrates. Among them, USP7’s stabilization of wild type p53 has garnered the most concern (11, 12). The p53 tumor suppressor, as the “guardian of the genome”, has been shown to regulate DNA repair, cell cycle arrest and apoptosis (13). However, over 50% of human cancers harbor mutant p53. p53 mutations not only lose its tumor-suppressor function, but also endow mutant p53 with a gain of function (GOF) (14). Like a oncogene, mutant p53 was involved in tumorigenesis, tumor progression, and chemotherapy resistance (14). Solomon et al. (15) demonstrated that mutant p53-expressing cell lines harbor larger sub-populations of cells highly expressing CSCs’ marker CD44, Lgr5, and ALDH. Thus, mutant p53 proteins may augment CCSCs phenotype and enhance colorectal tumorigenesis. Growing evidences suggested that deubiquitinating enzymes could regulate several signaling pathways and thus leading to survival of CSCs (16, 17). However, the biological function of USP7 on cancer stem cell expressing mutant p53 remains unexplored.

In this study, we showed that USP7 and mutant p53 were dramatically elevated in CSC-enriched colorectal cancer cells and USP7 expression was positively associated with self-renewal and maintenance of CCSCs. USP7 depletion significantly reduced proliferation of CRCs and suppressed the self-renewal of CCSCs. Further studies indicated that USP7 knockdown could significantly decrease mutant p53 protein levels both in CRCs and CSC-enriched colorectal cancer cells. Moreover, USP7 interacted with and stabilized mutant p53. Thus, inhibition of USP7 suppresses self-renewal and maintenance of CSCs. We postulate that USP7 may become a novel molecular target for p53-mutant cancers.

## 2 Materials and methods

### 2.1 Cell culture

The cell lines HCT116 and SW480 were purchased from ATCC; MDA-MB-468, Kasumi-1, H1299 and SKBR3 from Cellcook Bio-technology. Cells were cultured in different mediums: high glucose DMEM medium for SW480, H1299, MDA-MB-468, Kasumi-1 and SKBR3 cells; RPMI-1640 medium for HCT116 cells. All mediums (Biological Industries, Kibbutz Beit Haemek, Israel) were supplemented with 10% FBS (Biological Industries, Kibbutz Beit Haemek, Israel). Cells were incubated at 37°C with 5% concentration of CO_2_ in a humidified atmosphere.

### 2.2 Plasmids and cell transfection

Plasmids were transfected using Lipofectamine 3000 (Invitrogen, Camarillo, CA, USA) according to the manufacturer’s instructions. pcDNA3.1-human p53, various mutants were a gift from Dr. Min Lu (Ruijin Hospital affiliated with Shanghai Jiao Tong University School of Medicine). Flag-USP7 was previously generated in our laboratory. pX330_SpCas9_USP7 Exon 3 gRNA was a gift from Roger Woodgate (Addgene plasmid # 131257; http://n2t.net/addgene:131257; RRID: Addgene_131257).

### 2.3 Wound healing assay

Cells were incubated in 6-well plates. When cellular density reached 80-100%, cells were wounded with a 200 μL micro-pipette tip. The wound areas were washed three times with PBS. Then, fresh medium without FBS was added to the plates. The wound areas were photographed at 0 h and 48 h.

### 2.4 Immunoprecipitation assay

Firstly, the pretreated cells were lysed with IP lysis buffer(Beyotime, Shanghai, China) containing protease inhibitor (Beyotime, Shanghai, China), and the protein lysates were then immunoprecipitated with the primary antibody and incubated in 4°C overnight. Subsequently, 30-40 μL of protein A/G-agarose PLUS-Agarose (Santa Cruz Biotechnology, Dallas, TX, USA) were added. After 4-5 h incubation, the magnetic beads were washed with IP buffer for 4 times, then heated in 2X loading buffer at 100°C for 10 min. Samples were blotted and probed with indicated antibodies.

### 2.5 Sphere formation assay

Cells were seeded into Ultra-Low Attachment plate (Corning, Tewksbury, USA). The DMEM/F12 medium (Gibco laboratories, Grand Island, N.Y.) was added with 2% B27 (Gibco laboratories, Grand Island, N.Y.), 20 ng/mL EGF (Proteintech, Rosemont, IL), and 10 ng/mL bFGF (Proteintech, Rosemont, IL). After 7 days, the sphere formation efficiency was evaluated.

### 2.6 Cell viability assay and colony assay

Cell viability was determined by MTS assay. Briefly, 5 × 10^3^ cells were seeded in 96-well plates in triplicates and cultured overnight. Cells were treated with different concentrations of P5091 (Selleck, Houston, TX, USA) in the set time. Then, discard the old culture medium gently and added 100 µL medium which mixed with 20 µL CellTiter 96^®^ Aqueous One Solution Reagent (Promega, Madison, WI, USA). Cells were incubated at 37°C for 20 min–60 min. The optical density (OD) was measured at a wavelength of 492 nm using a microplate reader (Allsheng, Hangzhou, China). For colony assay, cells were seeded as single cells in 6-well plates and cultured for additional 7 days before colonies stained with crystal violet (Solarbio, Beijing, China). The number and volume of colonies were obtained under the microscope.

### 2.7 Western blot analyses

Cells were lysed on ice in RIPA buffer (Beyotime, Shanghai, China) containing protease inhibitor cocktail (Beyotime, Shanghai, China) for 40 min. Total proteins were quantified using a Pierce BCA Protein Assay Kit (Beyotime, Shanghai, China), and the lysates were diluted to approximately equal concentrations before heating in 5X loading buffer (Solarbio, Beijing, China). Samples were separated by 8-12% sodium dodecyl sulfate-polyacrylamide gel electrophoresis (SDS-PAGE), then transferred to PVDF membranes (Millipore, MA, USA), and blocked with 5% non-fat milk for 1-2 h at room temperature. The transferred PVDF membranes were incubated with the following primary antibody at room temperature for 3 h: USP7 and p53 (primary antibody: 1:500, Santa Cruz Biotechnology, Dallas, TX, USA), CD133, CD166, CD44 and GAPDH (primary antibody: 1:1000, Proteintech, Rosemont, IL). Finally, membranes were incubated with anti-IgG secondary antibodies (Proteintech, Rosemont, IL) for 1 h. The protein band were incubated with Pierce ECL substrate (Proteintech, Rosemont, IL) and visualized by images were captured with ChemiDoc™ MP imaging system (BioRad, Redmond, WA).

### 2.8 RNA extraction and real-time quantitative PCR analysis

Total RNA was extracted from cultured cells with EastepTM Total RNA Super Extraction Kit (Promega, Madison, WI, USA). In total, 1.5 μg of total RNA was reverse-transcribed with the GoScrip™ Reverse Transcription Mix, Oligo(dT) (Promega, Madison, WI, USA). The mRNA expression levels of USP7, p53, OCT4, SOX2 and NANOG were analyzed with the Easep®qPCR Master Mixs (2X) (Promega, Madison, WI, USA) according to the manufacturer’s instructions. The relative expression levels of mRNA were evaluated by using the 2−ΔΔCt method. PCR primer sequences were shown at **Table 1**.

**Table 1:** Primers for qRT-PCR.

Gene	Primer Sequence
human USP7	Forward: 5′-GTCACGATGACGACCTGTCTGT-3′
	Reverse: 5′-GTAATCGCTCCACCAACTGCTG-3′
human SOX2	Forward: 5′-TACCTCTTCCTCCCACTCC-3′
	Reverse: 5′-TGTGTGAGAGGGGCAGTGT-3′
human TP53	Forward: 5′-CCTCACCATCATCACACTGG-3′
	Reverse: 5′-GCTCTCGGAACATCTCGAAG-3′
human OCT4	Forward: 5′-GTGGAGGAAGCTGACAACAA-3′
	Reverse: 5′-ATTCTCCAGGTTGCCTCTCA-3′
human NANOG	Forward: 5′-CAGTCCCAAAGGCAAACAA-3′
	Reverse: 5′-CTGCTGGAGGCTGAGGTAT-3′
Human GAPDH	Forward: 5′-GGACCTGACCTGCCGTCTAG-3′
	Reverse: 5′-GTAGCCCAGGATGCCCTTGA-3′

### 2.9 Statistical analysis

Data are expressed as mean ± SD. Statistical comparisons between groups were conducted by unpaired Student’s t-test. * indicates p < 0.05; ** indicates p < 0.01; and *** indicates p < 0.001. p < 0.05 was considered to be statistically significant.

## 3 Results

### 3.1 USP7 is highly expressed in colorectal cancer stem-like cells

**Figure 1:**
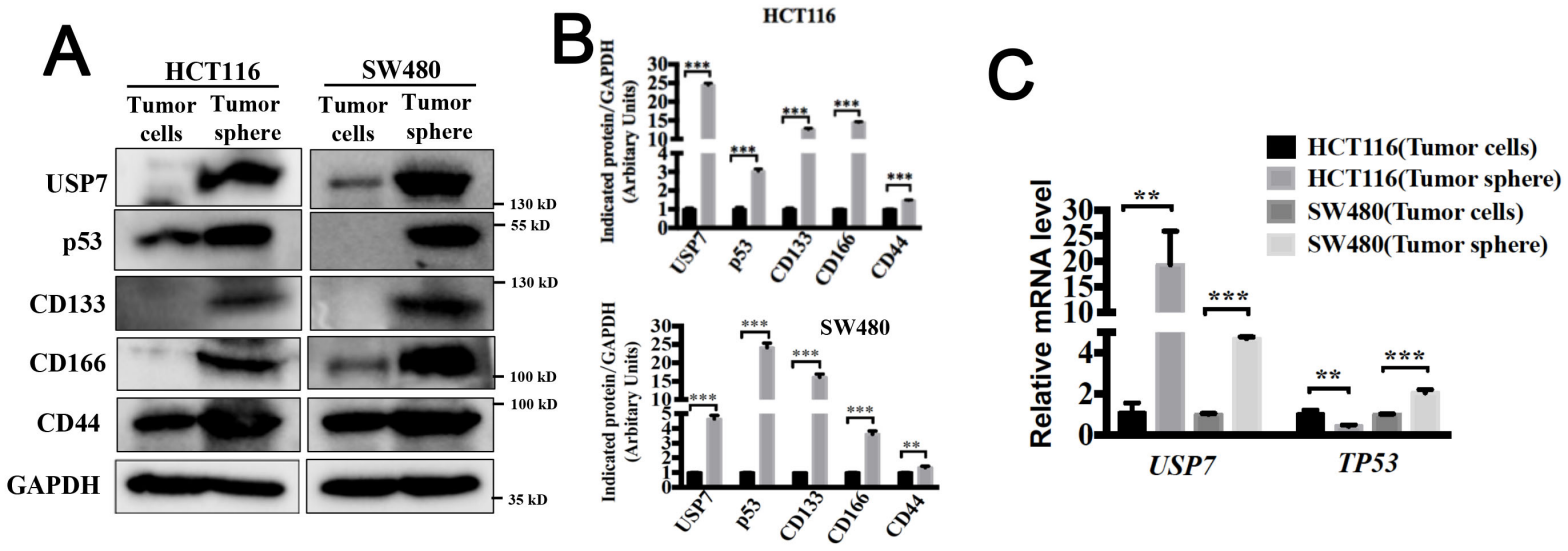
USP7 is highly expressed in colorectal cancer stem-like cells. **(A)** Protein expression levels of USP7, p53, CD133, CD166 and CD44 were tested in spheroid cells and adherent cells by western blotting. **(B)** Quantification of indicated protein levels in **(A)** by NIH ImageJ software. **(C)** mRNA expression levels of USP7 and TP53 were determined by qPCR. All values represented the mean ± SD (n = 3).The significance was determined by student’s t test (**P< 0.01 and ***P< 0.001 vs. control).

USP7 has been previously confirmed to overexpress in colorectal cancer cells and neoplastic tissues (18). We next compared the protein level of USP7 between colorectal cancer stem-like cells and their counterpart adherent cells. Spheroid formation model has been suggested to be an essential tool for confirming CSC-like features *in vitro* (19). HCT116 endogenously expressing wild type p53 and SW480 harboring p53 point mutations (R273H/P309S) were used for the following experiments. As shown in **Figures 1A, B**, USP7 protein level and the stem cell markers CD133, CD166 and CD44 were significantly higher in spherical cells compared to their counterpart adherent cells. Additionally, the mRNA level of USP7 was elevated in SW480 and HCT116 spherical cells (**Figure 1C**). We also determined the amount of mutant p53 that promotes acquisition of CSCs features (15). We observed a higher protein (**Figure 1A**) and mRNA level (**Figure 1C**) of mutant p53 in CCSC enriched tumor spheres. The tumor-suppressor p53 was found to ensure the genomic stability of stem cells (13). In our study, we observed a lower mRNA level of wild type p53 in spheres (**Figure 1C**), but the protein levels were increased (**Figures 1A, B**). Taken together, these results indicated that USP7 was highly expressed in CSC-enriched colorectal cancer cells.

### 3.2 P5091 reduces the population of CSCs and wound healing capacity of colorectal cancers

**Figure 2:**
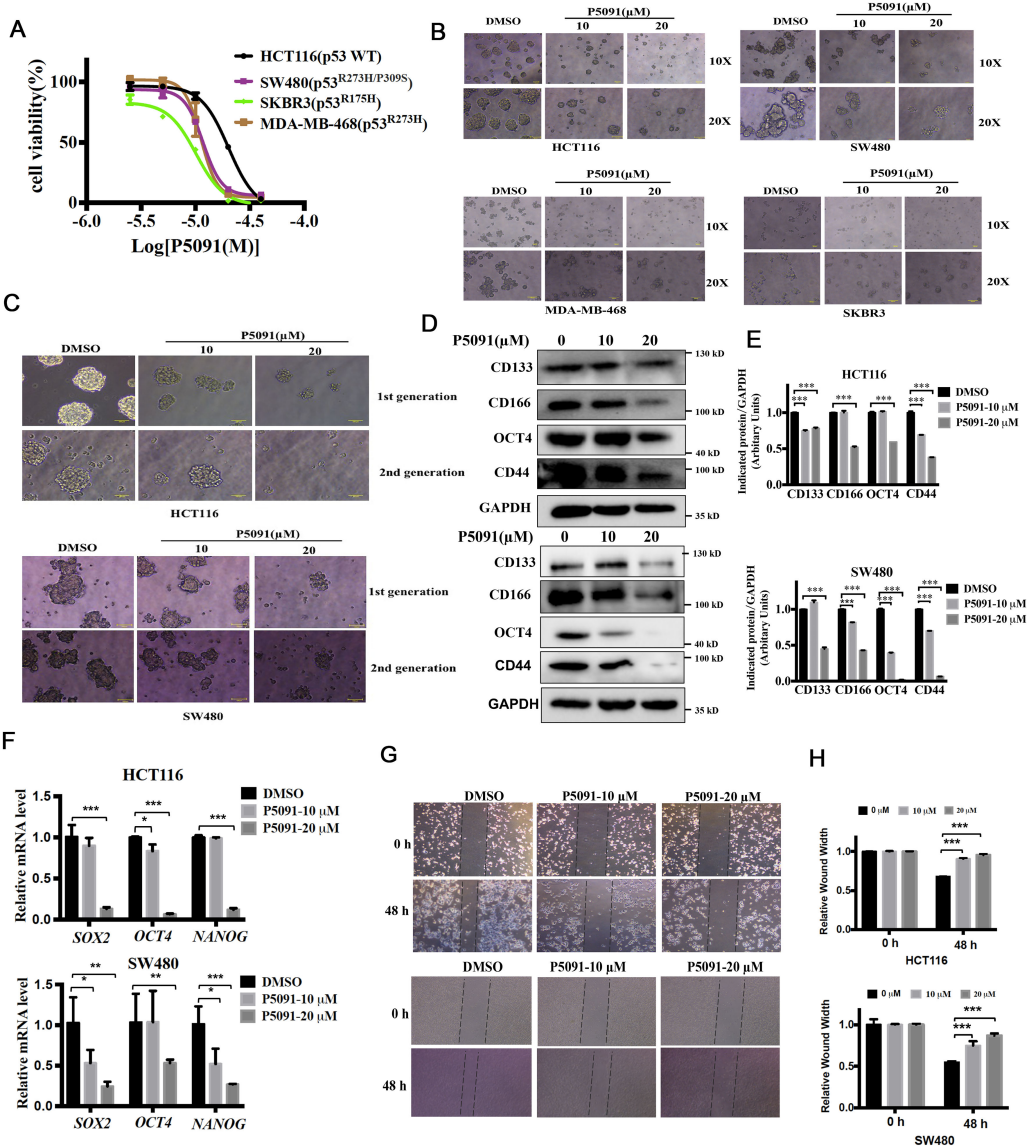
P5091 reduces the population of CSCs and inhibits cell migration capacity of colorectal cancers. **(A)** MTS assay were used to determine the growth inhibition effect of P5091 on cancer cells. **(B)** Cells were cultured under ultralow-attachment conditions for 7 days to enrich CSCs, and then treated with P5091 for 24 h (original magnification, 10× or 20×). **(C)** The spheroids size of 1st spheroid formation (upper) and 2nd spheroid formation (down) of HCT116 and SW480 cells were determined (original magnification, 20×). **(D)** The protein levels of CD133, CD166, CD44 and OCT4 were determined via western blot. **(E)** Quantification of indicated protein levels in **(D)** were detected by NIH ImageJ software. The values represented the mean ± SD (n = 3). **(F)** The mRNA levels of *SOX2*, *OCT4* and *NANOG* were determined by qPCR, GAPDH mRNA was used as an internal control. **(G, H)** Wound closure of HCT116 and SW480 cells in the presence or absence of P5091. Data are shown as means ± SD. The significance was determined by student’s t test (*P < 0.05, **P < 0.01 and ***P < 0.001 vs. control).

USP7 is a key regulator of the p53 pathway by stabilizing MDM2 which is a negative regulator of p53 (11, 12). Many compounds have been reported to inhibit USP7 activity and suppress cancer cell growth via activating p53 in cancer cells with wild type p53 (20). However, the biological function of USP7 on cancer cells which expressing mutant p53 remain unknown. First, HCT116 endogenously expressing wild type p53 and SKBR3, SW480, MDA-MB-468, which endogenously expressing a single copy of TP53 gene with R175H and R273H mutations, respectively, were used to evaluate the effects of P5091 on tumor cell proliferation. As illustrated in **Figure 2A**, P5091 showed antiproliferative activities in both p53 wild-type and mutant cancer cells. Next, we examined whether P5091 effectively inhibited CSCs with hot-spot mutations of p53. Cells spheroid were treated with P5091 for 24 h, and the effect of P5091 on CSCs was determined. As shown in **Figure 2B**, P5091 significantly impaired the spheroid formation abilities, including spheroid sizes and numbers, indicating the attenuated generation of CSCs and self-renewal capabilities. Next, we focused on colorectal cancer cells. The self-renewal capabilities of CCSCs were assessed by 1st and 2nd spheroid formation assay. The results showed that P5091 significantly decreased spheroid sizes and numbers of HCT116 (p53 WT) and SW480 (p53 R273H/P309S) cells in a dose-dependent manner (**Figure 2C**). Furthermore, western blot results showed that P5091 treatment decreased the protein levels of CCSCs markers, such as CD133, CD166, CD44 and OCT4 (**Figures 2D, E**). Likewise, P5091 also reduced the expression of stemness-related genes including *SOX2*, *NANOG* and *OCT4* of HCT116 and SW480 cells (**Figure 2F**). The epithelial-mesenchymal transition (EMT) also plays crucial roles in stem cell differentiation (21). As expected, P5091 treatment significantly reduced wound healing capacity of HCT116 and SW480 cells (**Figures 2G, H**). These results revealed that inhibition of USP7 might inhibits stemness and cell growth of colorectal cancers with wild-type or mutant p53.

### 3.3 USP7 depletion suppresses the stemness of colorectal cancer stem-like cells

**Figure 3:**
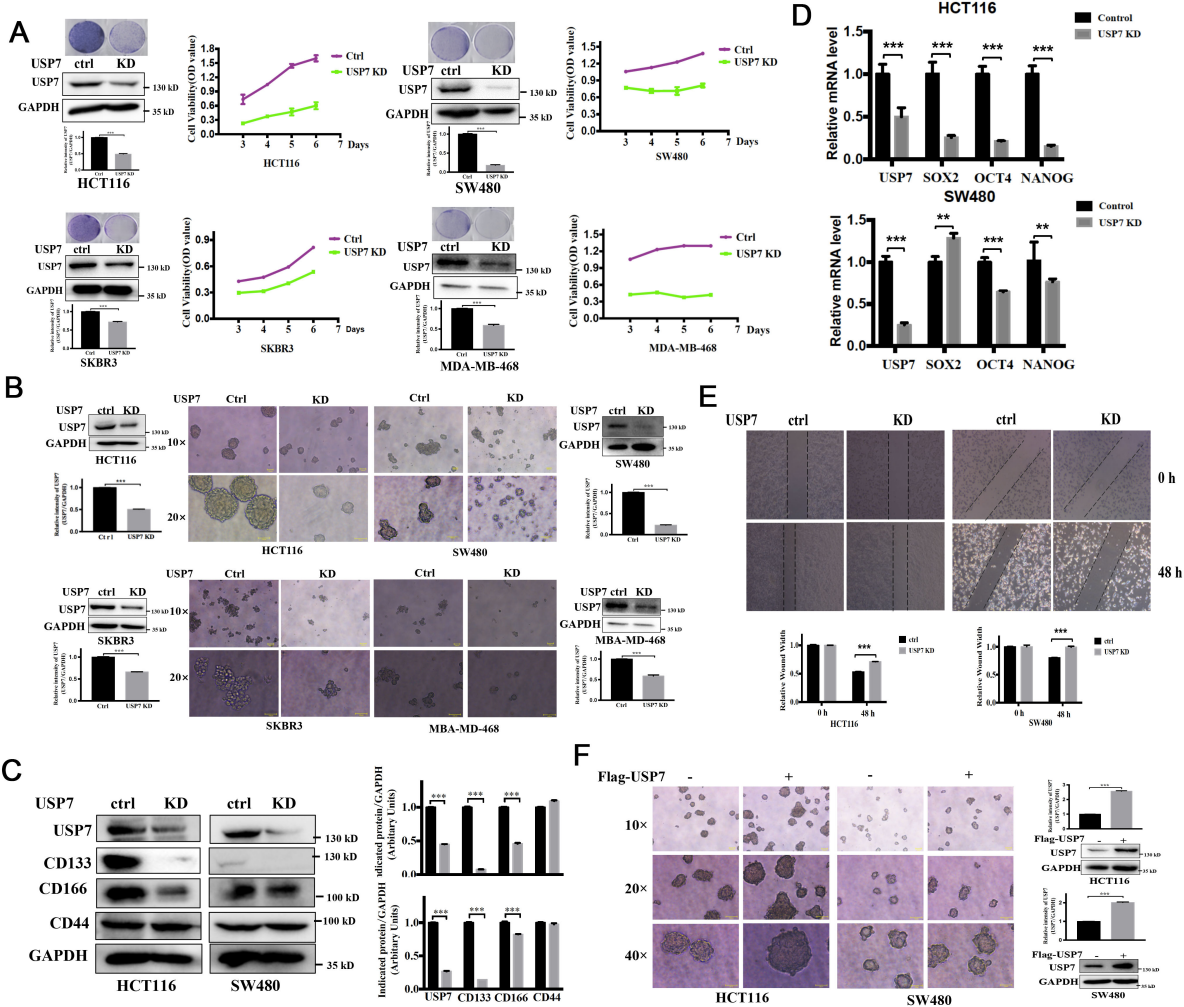
Downregulation of USP7 weakens cell self-renewal of CSCs. **(A)** Growth curves depicting the slow proliferation of HCT116, SW480, SKBR3 and MDA-MB-468 cells with USP7 interference. Cell proliferation was assessed by MTS assay and colony formation assay. USP7 protein levels were confirmed by western blot. **(B)** Comparison of sphere formation the absence or presence of USP7. (original magnification, 10× or 20×). USP7 protein levels were confirmed by western blot. The USP7 and GAPDH blots of MDA-MB-468 between **(A, B)** were reused. **(C)** The protein levels of CD133, CD166 and CD44 were determined via western blot. **(D)** The mRNA levels of *USP7, SOX2, OCT4* and *NANOG* were determined by qPCR. GAPDH mRNA was used as an internal control. **(E)** Wound closure among normal and USP7 knockdown cell lines at 48 h **(F)** Comparison of sphere formation among normal and USP7 overexpression cell lines (original magnification, 10×, 20× and 40×). USP7 levels were tested by western blot. Quantification of all indicated protein levels by NIH ImageJ software. The values represented the mean ± SD (n = 3). The significance was determined by student’s t test (**P < 0.01 and ***P < 0.001 vs. control).

Chang et al. (8) reported that USP7 protein level was elevated in HCT116 and HT29 cells, particularly in the CSCs. In this study, we focused on the effects of USP7 on cancer cells with different p53 status. First, we use plasmid to knockdown USP7 in HCT116, SW480, SKBR3 and MDA-MB-468 cells. USP7 protein levels significantly decreased with knockdown plasmid (**Figure 3A**). Previous study has reported that knockdown of USP7 significantly inhibited HCT116 cell proliferation (18). For cancer cells with different p53 status, our results showed that USP7 knockdown not only suppressed cell proliferation and colony formation of HCT116, but also suppressed SW480, SKBR3 and MDA-MB-468 cells growth (**Figure 3A**). In addition, like P5091, USP7 knockdown significantly inhibited the formation of spheroids, as well as their sizes and volumes (**Figure 3B**). Then, we focused on colorectal cancer cells. As showed in **Figure 3C**, western blot results demonstrated that USP7 knockdown decreased the protein levels of CSC markers, such as CD133 and CD166, but no on CD44. Likewise, USP7 depletion also reduced the expression of stemness-related genes *NANOG* and *OCT4* (**Figure 3D**). USP7 depletion also significantly reduced wound healing both in HCT116 and SW480 cells (**Figure 3E**). Furthermore, the size and volume of spheroids were increased in USP7 overexpression plasmid transfected HCT116 and SW480 cells (**Figure 3F**). Collectively, these data indicated that downregulation of USP7 was involved in the attenuation of the CSCs stemness and wound healing capacity which cells with wild-type or mutant p53.

### 3.4 Knockdown of USP7 attenuated the mutant p53

**Figure 4:**
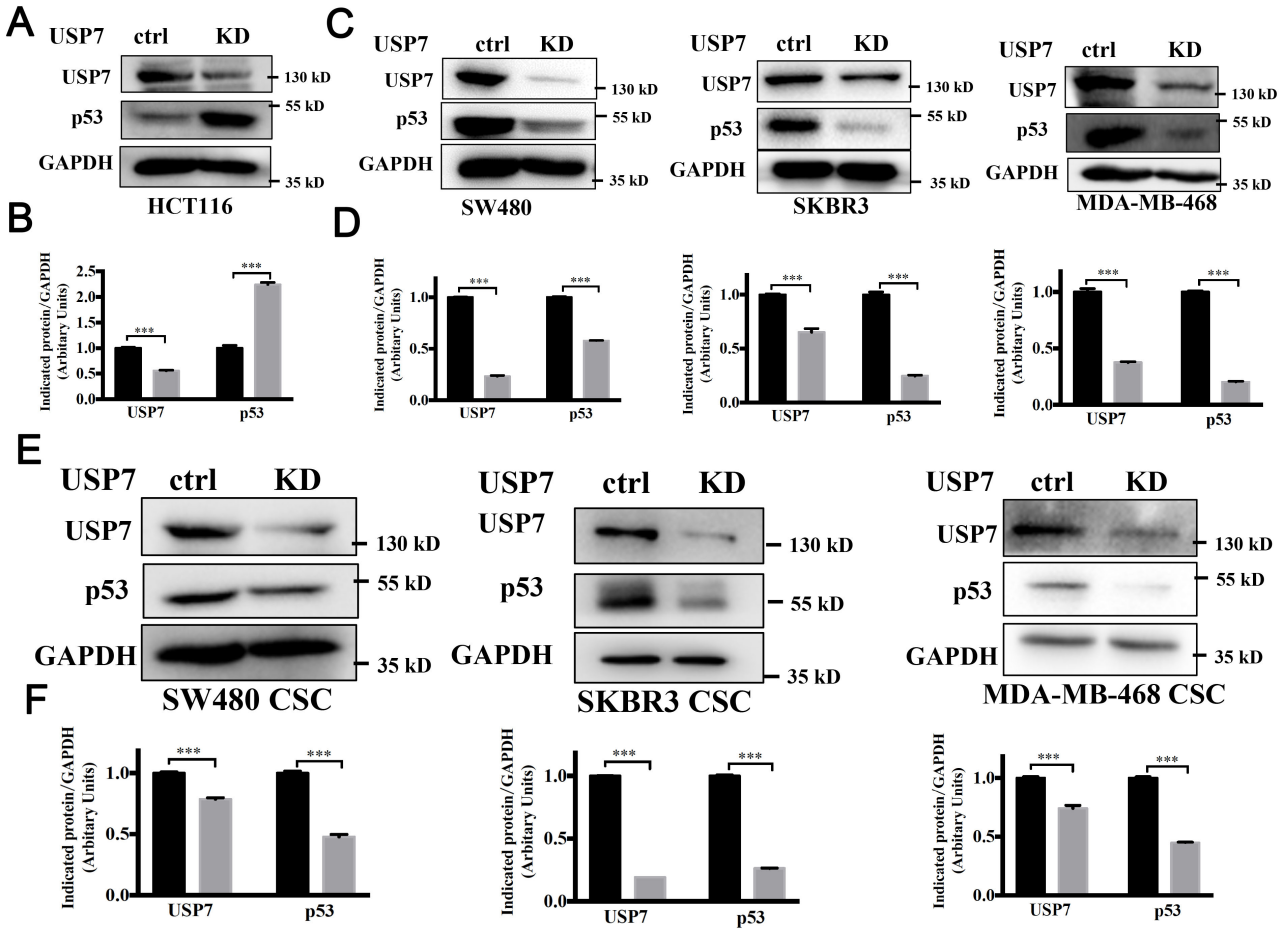
Knockdown of USP7 attenuated the mutant p53. **(A–D)** Knockdown of USP7 induced levels of wild-type p53 **(A, B)** and reduced levels of mutant p53 **(C, D)**. The USP7 and GAPDH blots of SKBR3 between **Figure 3A** and **(C)** were reused. **(E, F)** Cells were transfected with USP7 knockdown plasmid for 48 h, then cultured under ultralow-attachment conditions for 7 days to enrich CSCs. USP7 and p53 protein levels were confirmed by western blot. The significance was determined by student’s t test (***P< 0.001 vs. control).

**Figure 5:**
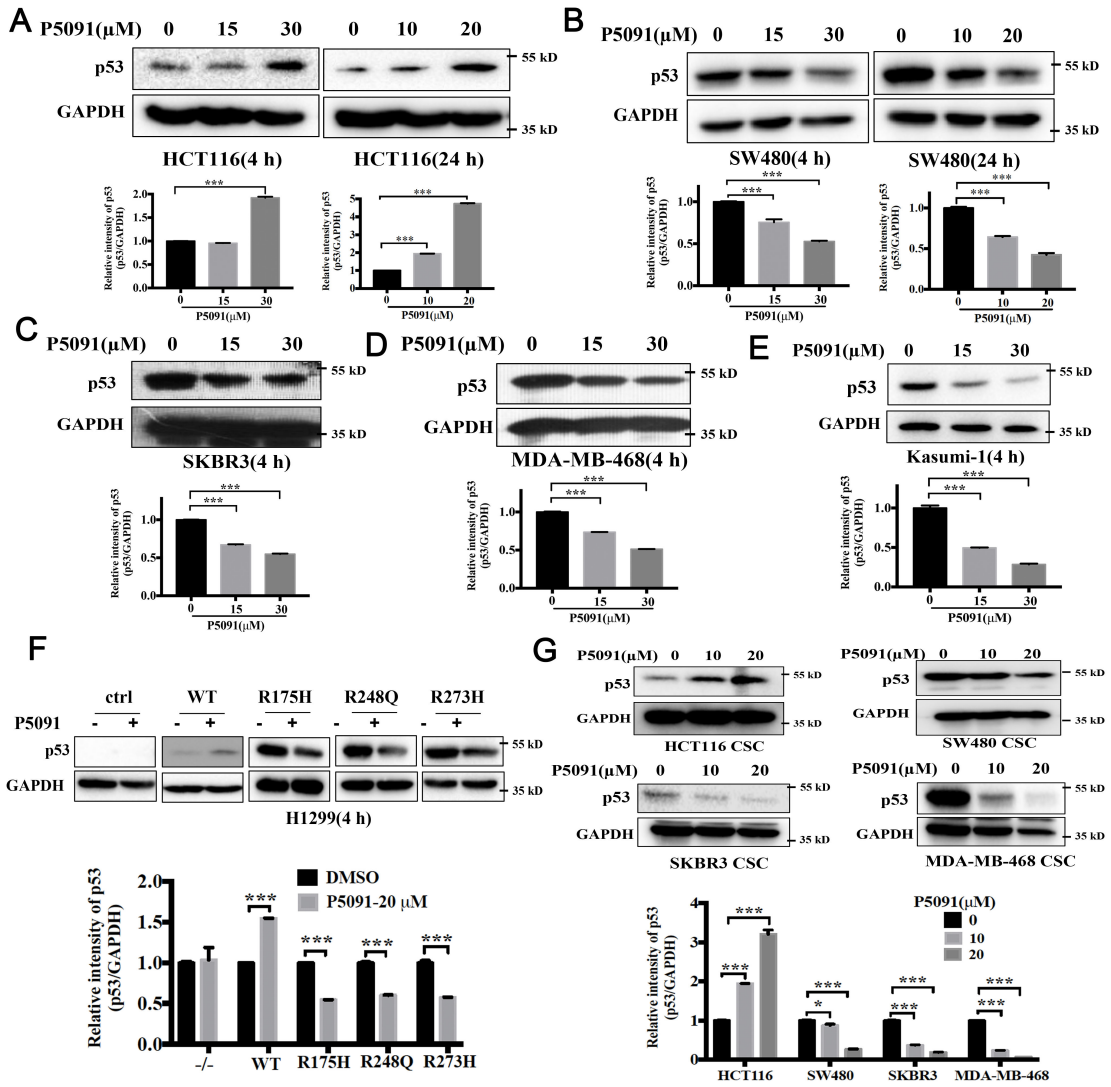
Pharmacologically blocking USP7 activity by P5091 attenuated the mutant p53. **(A–E)** P5091 treatment dose-dependently increased the level of wild-type p53 **(A)** and reduced levels of mutant p53 **(B–E)**. **(F)** P5091 treatment reduced level of ectopically expressed mutant p53. H1299 cells were transiently transfected with p53 expression vectors. P53 protein levels were confirmed after 4 h treatment with P5091. **(G)** Cells were cultured under ultralow-attachment conditions for 7 days to enrich CSCs following 4 h treatment with P5091. Western blotting was used to evaluate the levels of p53. Quantification of all indicated protein levels were obtained by NIH ImageJ software. The values represented the mean ± SD (n = 3). The significance was determined by student’s t test (*P < 0.05 and ***P < 0.001 vs. control).

Multiple studies have shown that USP7 depletion promotes the degradation of MDM2, activates the p53 signaling, and causes cell cycle arrest and apoptosis (22–24). Mutant p53 is considered to control the self-renewal of CSCs in colorectal cancers (25). Above results proved that knockdown or inhibition of USP7 could inhibit the self-renewal of CSCs with different p53 mutations. Therefore, we postulate that USP7 possibly affects mutant p53 to affect stem cell property in CRCs. The mutant p53 levels in USP7 knockdown cells were tested by western blotting assays. As expected, wild-type p53 levels were increased in USP7 knockdown HCT116 cells (**Figures 4A, B**). Interesting, when knocking down USP7 in mutant p53-expressing cancer cells the p53 protein levels were dramatically decreased (**Figures 4C, D**). Similar date were obtained for the spheres (**Figures 4E, F**). Likewise, wild-type p53 levels were dramatically increased after USP7 inhibitor P5091 treatment (**Figure 5A**), while mutant p53 levels were decreased in cancer cell endogenously expressing mutant p53 (**Figures 5B–E**). Furthermore, human p53-null cancer H1299 cells were transfected with p53 (WT, R175H, R248Q and R273H) expression vectors. Western blot results showed that wild type p53 level was increased, but all three variants of mutant p53 proteins were significantly reduced following P5091-treated (**Figure 5F**). P5091 treatment also increased wild type p53 protein level, and reduced the mutant p53 levels of spheroid (**Figure 5G**). These data suggested that USP7 might stabilize mutant p53 in p53-mutant expressing cancer cells.

### 3.5 USP7 binds to and promotes stabilization of mutant p53

**Figure 6:**
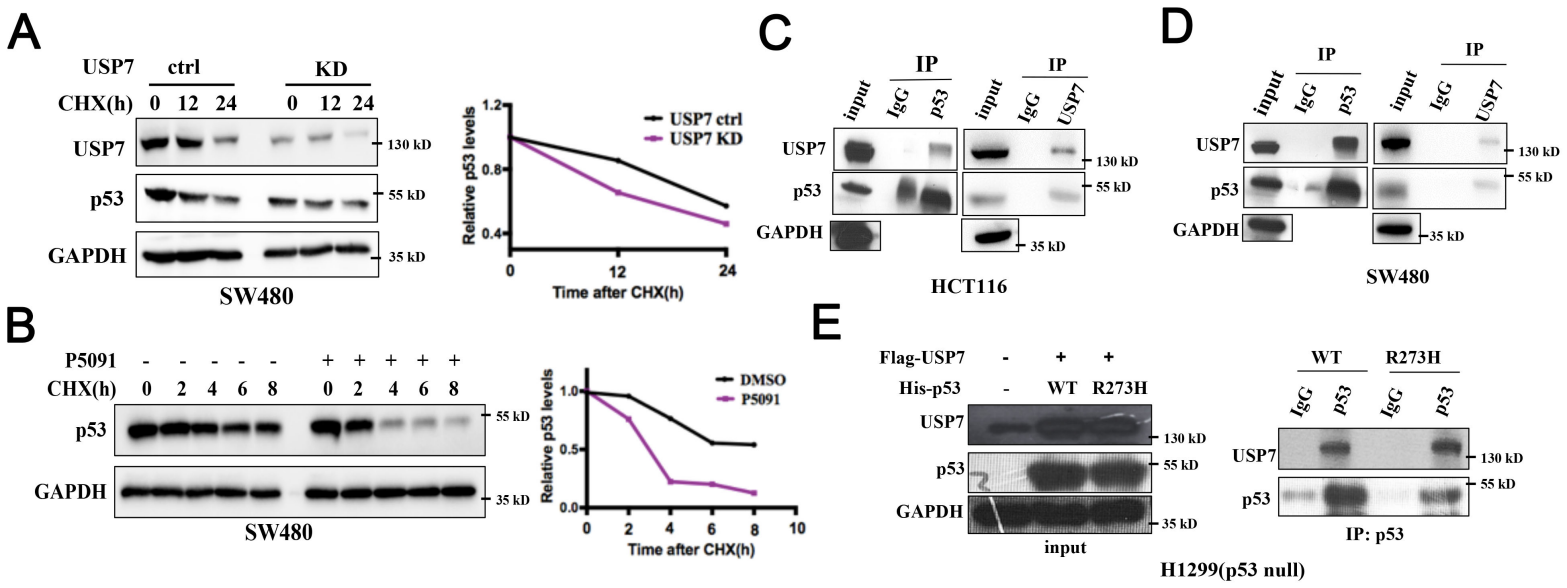
USP7 binds to and promotes stabilization of mutant p53. **(A)** Cells were transfected with USP7 knockdown plasmid for 48 h, then treated with CHX for the indicated times. **(B)** SW480 cells were pretreated with DMSO or P5091 for 2 h, then added 100 µg/mL CHX for the indicated times. Mutant p53 levels were detected by western blot assay. **(C, D)** The interaction effects of endogenous USP7 with wild-type and mutant p53 were observed in human colorectal cancer cell HCT116 **(C)** and SW480 **(D)**. **(E)** Ectopically expressed USP7 preferentially interacted with not only wild-type p53 protein but also mutant p53 (R273H) protein in H1299 cells. H1299 cells were transiently transfected with vectors expressing USP7 together with human wild-type p53 or mutant p53 (R273H) expression vectors.

It was reported that USP7 interacts with and stabilizes wild-type p53 (26). However, the role of USP7 on mutant p53 proteins has not been well-explained. We next detected whether USP7 could also regulate mutant p53 stability. The cycloheximide (CHX) chase assay was used to assess the effect of USP7 on mutant p53. As shown in **Figure 6A**, knockdown of USP7 significantly decreased the stability of mutant p53 in SW480 cells. Likewise, USP7 inhibitor P5091 also led to similar results (**Figure 6B**). Next, human colorectal cancer HCT116 and SW480 cell lines were used to investigate the interaction between the endogenous USP7 and p53 proteins. Co-IP assays employing either anti-p53 or anti-USP7 antibodies showed that USP7 interacted with not only wild-type p53 (**Figure 6C**) but also mutant p53 (**Figure 6D**). Additionally, human p53-null cancer H1299 cells were transfected with USP7 expression vectors together with human wild-type p53 or mutant p53 (R273H) expression vectors. Co-IP assays showed that USP7 preferentially bound to both wild-type and mutant p53 (**Figure 6E**). Together, these data suggest endogenous USP7 may bind to and regulate mutant p53 protein stability.

## 4 Discussion

DUBs is considered as a rational target for the first-in-class medicines development (27). Among all DUBs, USP7 gets more attention because of its involvement in multiple oncogenic pathways (28). It has been reported that USP7 significantly increased in the CRC cells and tissues, particularly in the CSCs (8, 18). Our data suggested that USP7 was highly associated with CSC properties. Knockdown of USP7 in colorectal cancer cells suppressed spheres formation and reduced cancer stem markers. It has been reported that USP7 deubiquitinates and stabilizes SOX2 thereby promoting mouse embryonic stem cells pluripotency (9). In our study, we demonstrated that USP7 was overexpressed in colorectal stem-like cancer cells. Knockdown of USP7 might reduce the mRNA levels of some CSCs-related genes, such as *OCT4* and *NANOG*. Our data further indicated that USP7 depletion attenuates the stemness in HCT116 and SW480 cells via inhibiting the protein levels of CCSCs-related proteins, including CD133 and CD166. In summary, we suggested that USP7 regulated the stemness and potentially acted as a critical marker of CSCs in colorectal cancers.

Wild-type p53 as well as MDM2 have been identified as substrates of USP7 (29). Previous study demonstrated that USP7 silencing was shown to increase the stability of p53 level by promoting MDM2 degradation (29). Interestingly, this phenotype was observed only in cancer cells expressing wild-type p53. TP53 is the most commonly mutated gene in human cancers (30). When mutated, p53 not only lose its wild-type p53 tumor suppressor function, rather it gains new functions that contribute to oncogenic properties (31). Colorectal cancer have the highest prevalence of p53 mutations, about 40% of CRCs harboring p53 mutations (32). The most frequently mutated codons in p53 are R175, R248, and R273 (32). Some studies reported that wild type p53 is a barrier for cancer cell formation (33). However, recent studies identified the GOF activities of mutant p53 in the acquisition of CSCs features (15, 25). In this study, we analyzed the effects of USP7 on cancer cells and cancer stem-like cells with different p53 status. USP7 depletion not only suppressed cell proliferation and colony formation of HCT116 harboring wild-type p53, but also suppressed SW480, SKBR3 and MDA-MB-468 harboring mutant p53. Wild type p53 has a short half-life, while mutant p53 accumulate to high levels in cancer cells (34). Therefore, accelerating mutant p53 degradation is an important therapeutic strategy (34). Our study found that USP7 knockdown could increase wild-type p53 protein level, which is consistent with the reported result (35). Meanwhile, we revealed for the first time that USP7 deplete could promote the degradation of mutant p53. In addition, compounds were discovered and proved to accelerate mutant p53 degradation. For example, gambogic acid has the ability to reduce the protein level of mutant p53 by preventing the formation of HSP90/mutant p53 complexes (36). Histone deacetylase (HDAC) inhibitor SAHA combined with the HSP90 inhibitor 17AAG could deregulate mutant p53 and arrest the growth of xenografts (37). Our results showed that P5091, a well-known USP7 inhibitor, could dramatically reduce the protein levels of mutant p53. Besides, mass spectrometry analysis found that USP7 was a p53-interactive protein (11). Our study confirmed that USP7 not only binds to and stabilizes wild-type p53, but also mutant p53. In a word, our study demonstrated that targeting USP7 represented a viable clinical strategy to kill tumors harboring mutant p53. As a deubiquitinase, USP7 is involved in the regulation of multiple proteins by deubiquitination, such as C-Myc, β-Catenin and NOTCH1 (38). However, our study found that USP7-mediated mutant p53 stabilization was not through deubiquitination (data not shown). Further study need to explore the degradation mechanism of USP7 on mutant p53.

Accumulating evidence indicates that the expression of USP7 is abnormally high in various cancer cells (10). As a druggable target for cancer therapy, many molecules have been found to target USP7 in recent years (20). P5091 was discovered in a high-throughput screening with an EC50 value of 4.2 μM against USP7 (23). Further study showed that P5091 triggered cytotoxicity to multiple myeloma cells is associated with activation of the Mdm2-p53-p21 signaling axis (23). Additionally, P5091 also inhibited the proliferation of colorectal cancer cells and breast cancer cells (18, 39, 40). However, there are no reports about P5091 regulating cancer stem cells. In our research, with sphere formation experiments, we verified that P5091 inhibited the formation of CSC-enriched cancer cells with wild-type or mutant p53. Inhibition of USP7 by P5091 significantly decreased spheroid sizes and numbers of colorectal cancer cells in a dose-dependent manner. P5091 treatment also decreased the expression of colorectal cancer stem cell markers. Moreover, P5091 strongly decreased mutant p53 protein levels in colorectal cancer cells and CSC-enriched colorectal cancer cells. Our study provides a possible therapeutic strategy in that treatment with a USP7 inhibitor would be useful for killing cancer cells through targeting mutant p53 degradation.

## Data Availability

The original contributions presented in the study are included in the article/supplementary material. Further inquiries can be directed to the corresponding author.
